# Preparation of Ovalbumin/Xanthan Gum/Chitosan Pickering Emulsion Oleogel Added with *Amomum villosum* Lour. Extract and Its Application in Cookies

**DOI:** 10.3390/gels10110683

**Published:** 2024-10-23

**Authors:** Shan Xue, Jilong Zhao, Zhouyi Xiong, Jie Huang

**Affiliations:** 1 College of Biological Science and Technology, Minnan Normal University, Zhangzhou 363000, China; zjl24@mnnu.edu.cn; 2 Research Institute of Zhangzhou-Taiwan Leisure Food and Tea Beverage, Zhangzhou 363000, China; 3 Zhangzhou Food Science Research Institute, Zhangzhou 363000, China; 4 School of Life and Health Technology, Dongguan University of Technology, Dongguan 523808, China; xiongzhouyi@dgut.edu.cn; 5 College of Chemistry, Chemical Engineering and Environment, Minnan Normal University, Zhangzhou 363000, China; hj1745@mnnu.edu.cn; 6 Fujian Provincial Key Laboratory of Modern Analytical Science and Separation Technology, Zhangzhou 363000, China

**Keywords:** *Amomum villosum* Lour. extract (AVE), ovalbumin/xanthan gum/chitosan Pickering emulsion oleogel, preparation, physicochemical properties, cookie application

## Abstract

In this study, a new oleogel system was constructed and used as a fat substitute in the processing of cookies. The preparation process of *Amomum villosum* Lour. extract (AVE) was optimized based on antioxidant activity and yield firstly. Then, the AVE, ovalbumin, chitosan, and xanthan gum were used as raw materials to prepare a composite Pickering emulsion oleogel. The results showed that when the concentration of AVE, chitosan, and XG were 0.1%, 2.5%, and 0.3%, respectively, a stable and uniformly distributed Pickering emulsion oleogel was formed. In this case, the particle size of the composite oleogel was relatively small; the absolute value of zeta potential was higher; the microstructure was more stable, with less aggregation and flocculation; and the thermal stability and freeze–thaw stability were excellent. In addition, the addition of AVE enhanced the gel properties of the oleogel and had good solid-like properties, and strengthened the binding force, as well as the oxidation stability, making the whole system more stable. In addition, the results of the application of the composite oleogel in the cookies showed that the AVE–ovalbumin/xanthan gum/chitosan Pickering emulsion oleogel had similar sensory and texture properties to the butter group. The addition of AVE can delay the crispness, cohesiveness, hardness, and the rate of malondialdehyde formation in cookies during storage. In conclusion, the AVE–ovalbumin/xanthan gum/chitosan Pickering emulsion oleogel had good physicochemical stability and showed great potential in replacing saturated fat (butter) in baking products (cookies).

## 1. Introduction

Traditional solid fats are widely used in bakery products to impart special flavor, texture, and taste characteristics [1]. However, most solid fats are made from hydrogenated vegetable oils, which contain more saturated fatty acids and trans fatty acids [2], and excessive intake causes harm to human health, thereby increasing the risk of diabetes, obesity, and other diseases [3]. Therefore, the food industry needs to develop a new type of alternative that not only has the structure and properties of traditional solid fats but also improves the fatty acid composition in foods.

Oil gelation is a healthy method. Liquid oil contains a large amount of unsaturated fatty acids, which can be converted into a solid gel without chemical modification. Emulsion oil gelation was established by using hydrophilic polymers, which made it possible to gelate liquid oils with food-approved hydrophilic polymers [4]. For some food-grade polysaccharides and proteins, these hydrated polymers can form gels in water. Through the solubility characteristics of hydrocolloids, hydrocolloids can be prehydrated first to form colloidal particles to stabilize the emulsion, and then, liquid oil can be wrapped in the grid structure formed by the polymer, thus forming food properties similar to traditional solid oils, as an alternative to trans fatty acids [5]. Oleogels are considered the “fat of the future” and can replace traditional solid fats in food applications, such as baking products, meat products, chocolate, candy, and certain spreads, and other food processing applications have been studied [1,3].

Ovalbumin is a spherical monophosphoglycoprotein with a molecular weight of 42–47 kDa, which is the most abundant protein in egg whites (54–60%) and plays a leading role in the foaming and gelation process of egg white proteins [6,7]. Ovalbumin has good emulsification and gelling properties and has been widely used to prepare nanoparticles or nanoemulsions to improve its water solubility, stability, and antioxidant activity and to deliver bioactive substances [8]. Chitosan is a derivative of chitin. Chitosan can be used as a Pickering stabilizer by forming particles through self-aggregation, ionic gels, polyelectrolyte composites, hydrophobic modification, etc. The formation of autopolymers can enhance hydrophobicity and thus oil affinity [9]. Huang et al. [10,11] showed that under certain conditions, ovalbumin can form an ordered spatial structure with chitosan, agaric gum, and other polysaccharides, effectively improving the stability of the protein–polysaccharide system. Xanthan gum, also known as Hansheng gum, is an extracellular microbial polysaccharide produced by the fermentation of sugar by Xanomonas bacterium. It has a variety of functions and can be used as a film-forming agent, gel thickening agent, stabilizer, etc., and is widely used in various fields [12]. Studies have shown that the complex polysaccharide and protein system can have better structural properties and stability than a single polysaccharide or polysaccharide system, and an orderly three-position network structure can be formed between molecules to better accommodate liquid fats [13]. For example, ovalbumin–xanthan gum [7] and ovalbumin–chitosan [10] can both form a stable emulsion system. Therefore, based on the above analysis, we boldly speculate that ovalbumin, chitosan, and xanthan gum can form a stable complex system under certain conditions.

In addition, it should be noted that unsaturated fatty acids have many benefits, but they are also more prone to oxidation reactions. During storage, oil will automatically oxidize to produce primary oxidation products such as hydroperoxide, which are very unstable in nature and are extremely easy to decompose into small molecules such as hydrocarbons, aldehydes, and ketones, and these secondary oxidation products are an important cause of food deterioration [14,15]. Therefore, adding a certain amount of antioxidants can effectively delay the automatic oxidation process of oil and improve the storage life of lipid foods.

*Amomum villosum* Lour. is a perennial herb of the Cardamom genus in the ginger family, mainly distributed in the Fujian, Guangdong, Guangxi, and Yunnan regions of China. Amomum has a history of more than 1300 years. It is a kind of high-quality food material with the same origin as medicine and food. Many studies have shown that *Amomum villosum* Lour. is rich in a large number of bioactive ingredients (such as polyphenols, flavonoids, phenolic acids, etc.), which have many effects such as anti-oxidation, antidiarrhea, regulating qi, etc., especially in the treatment of gastrointestinal diseases [16]. Feng et al. showed that the alcohol extract of *Amomum villosum* Lour. could effectively inhibit *Escherichia coli*, *Staphylococcus aureus*, and *Bacillus subtilis*, and the main antibacterial effects were polyphenols and flavonoids in the alcohol extract. Different extraction conditions will significantly affect the extraction rate and composition of *Amomum villosum* Lour. extract (AVE) [17]. In addition, studies have shown that the addition of polysaccharide can improve the stability of ovalbumin–polyphenol (ferulic acid) emulsions [11]. However, at present, studies on the application of amomum extract and its gels in emulsions are scarce, so studies on the application and influence of AVE in complex protein–polysaccharide systems has theoretical value.

Based on the above analysis, this study first optimized the preparation process of AVE, and then prepared a composite Pickering emulsion oleogel added with AVE, using ovalbumin, chitosan, and xanthan gum as raw materials, and investigated the effects of different addition levels of raw material on the physicochemical properties of an AVE–ovalbumin/xanthan gum/chitosan Pickering emulsion oleogel. Finally, the composite oleogel was applied to the production of cookies as a fat substitute in order to provide a theoretical basis for the development and application of a new functional oleogel.

## 2. Results and Discussion

### 2.1. Optimization of AVE Preparation Process

**Figure 1:**
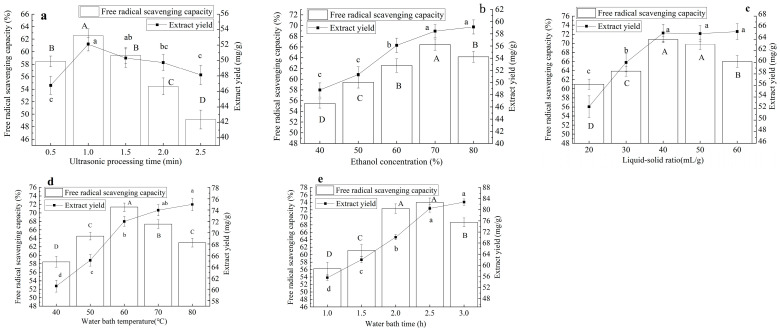
Effects of ultrasonic time (**a**), liquid–solid ratio (**b**), ethanol concentration (**c**), extraction temperature (**d**), and extraction time (**e**) on free radical scavenging capacity and extract yield of AVE. (The different letters A–D marked in the bar chart indicated significant differences in the results of free radical scavenging capacity (*p* < 0.05); The different letters a–e marked in the line indicated significant difference in extraction yield (*p* < 0.05)).

The effects of different ultrasonic times (a), liquid–solid ratios (b), ethanol concentrations (c), extraction temperatures (d), and extraction times (e) on the free radical scavenging capacity and extraction yield of AVE are shown in Figure 1. With the extension of the ultrasonic processing time, the absorption capacity of AVE to oxidizing free radicals first increased and then decreased (*p* < 0.05). When the ultrasonic time was 1 min, the scavenging rate of oxidizing free radicals had the maximum value. The change in AVE yield was generally consistent with the change in scavenging capacity, so the ultrasound time was determined to be 1 min (Figure 1a). Appropriate ultrasonic treatment can improve the extraction efficiency of active ingredients, but a too-high-intensity ultrasonic treatment will produce a strong cavitation effect and lead to the degradation of some macromolecular components [18].

With the increase in ethanol concentration, the scavenging activity of oxidative free radicals of AVE first increased and then decreased (*p* < 0.05), and a 70% ethanol concentration had the best effect, while the yield of AVE gradually increased with the increase in ethanol concentration, and there was no significant difference between the extraction yield of 70% and 80% ethanol extracts (*p* > 0.05). Therefore, taking comprehensive consideration, 70% ethanol was selected for the following experiment (Figure 1b). This may have been due to the fact that when the concentration of ethanol was too high, the polarities of polyphenols in *Amomum villosum* Lour. and solvents were greatly different, and the protein denatured, resulting in the decrease in the solubility of polyphenols. In addition, the dissolution of alcohol-soluble impurities and pigment components would also affect the extraction [19].

In the range of a liquid–solid ratio of 20~60 mL/g, with the increase in the liquid–solid ratio, the scavenging activity AVE to oxidizing free radicals showed a trend of first increasing and then gradually decreasing (*p* < 0.05). When the liquid–solid ratio was 40 mL/g, the scavenging rate had the maximum value, and there was no significant difference between the scavenging rate and that of 50 mL/g (*p* > 0.05). With the increase in the liquid–solid ratio, the extraction yield of AVE showed a trend of a gradual increase, and the yield difference was not significant in the range of 40~60 mL/g, because when the extracts reached a certain amount, the active components had been basically leached, while other substances such as polysaccharides and pigments were dissolved out at this time, which would have affected the antioxidant activity [20]. From the perspective of cost saving, the liquid–material ratio of 40 mL/g was selected (Figure 1c).

The scavenging activity and extraction yield of AVE were significantly affected by water bath temperature (*p* < 0.05). With the increase in temperature, the absorption capacity of AVE to oxidizing free radicals first increased and then decreased (*p* < 0.05). When the temperature of the water bath was 60 °C, the scavenging capacity was the highest, which may have caused the molecular structure of antioxidant components in AVE to change. In addition, a high temperature accelerated the oxidation and degradation of active ingredients, resulting in a decrease in extraction yield [20]. The extraction yield of AVE increased gradually with the increase in water bath temperature. When the temperature was 80 °C, the yield had a maximum value. After comprehensive consideration, 60 °C was selected for the follow-up test (Figure 1d).

In the range of 1~3 h water bath time, the free radical scavenging capacity of AVE first increased and then decreased with the extension of time. When the water bath time was 2.5 h, the scavenging rate had the maximum value, and there was no significant difference between the scavenging rate and that at 2 h (*p* > 0.05). The yield of AVE increased significantly with the extension of water bath time (*p* < 0.05). This may have been because the appropriate extension of the water bath time can make more effective components in the sand kernel dissolve out, but too long a heating time will also lead to the loss of some heat-sensitive effective components, structural damage, and waste of resources [21]. After comprehensive consideration, the extraction time was selected to be 2.5 h (Figure 1e). *Amomum villosum* Lour. is a kind of high-quality food with the same origin as medicine and food. It contains a large number of chemical active ingredients and has a variety of biological effects, mainly manifested in the protection of the gastrointestinal tract and antioxidant, hypoglycemic, immune regulation, and antibacterial activity and other aspects [22]. In this study, when the ultrasonic treatment time was 1 min, the ethanol concentration was 70%, the liquid–solid ratio was 40 mL/g, the extraction temperature was 60 °C, and the extraction time was 2.5 h, AVE had better free radical scavenging activity (72.34 ± 1.25) and a higher extraction yield (82.74 mg/g).

### 2.2. Effects of Addition of AVE, Chitosan, and Xanthan Gum on Physicochemical Properties of Pickering Emulsion Oleogel

#### 2.2.1. Effect of Addition of AVE, Chitosan, and Xanthan Gum on Particle Size and Absolute Value of Zeta Potential of Pickering Emulsion Oleogel

**Figure 2:**
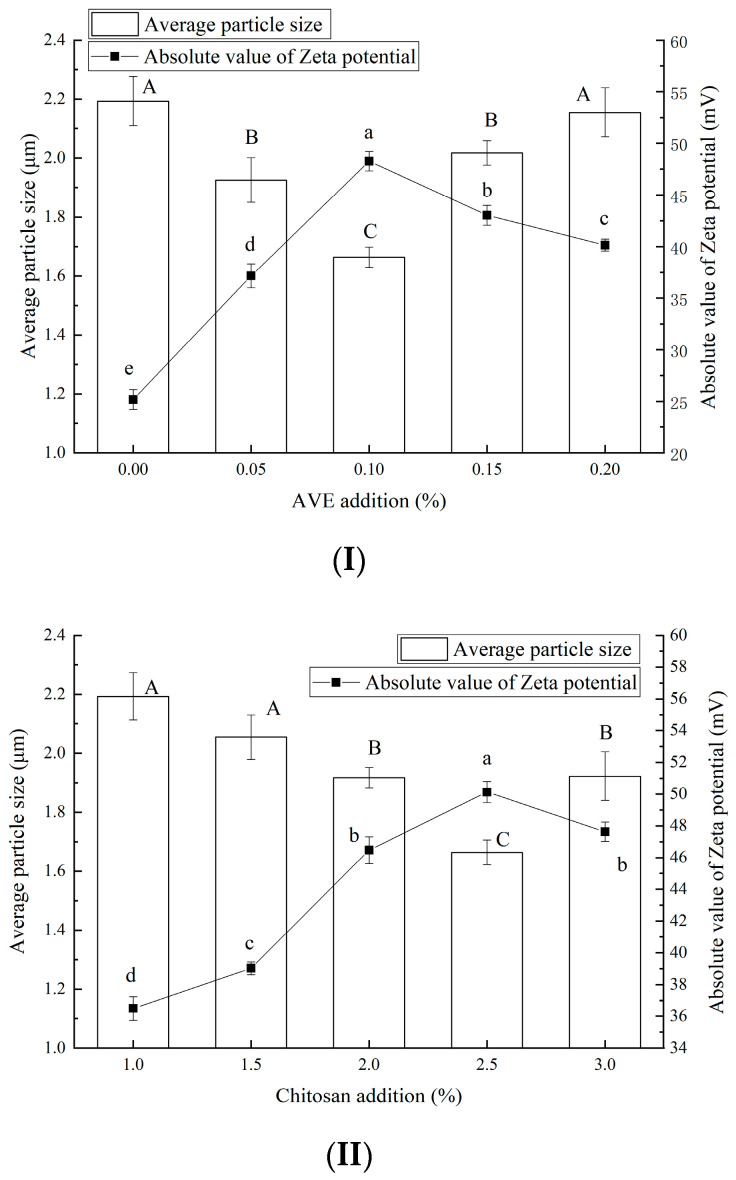

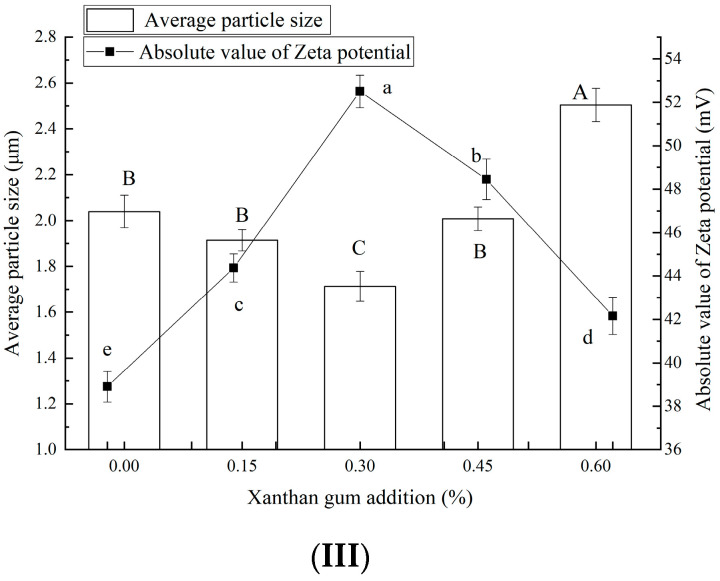
Effects of addition of AVE (**I**), chitosan (**II**), and xanthan gum (**III**) on particle size and absolute value of Zeta potential of Pickering emulsion oleogel (in the figure (**I**), a–e represent 0, 0.05%, 0.1%, 0.15%, and 0.2% additions of AVE, respectively. In the figure (**II**), a–e represent 1.0%, 1.5%, 2%, 2.5%, and 3% additions of chitosan, respectively. In the figure (**III**), a–e represent 0%, 0.15%, 3%, 0.45%, and 0.6% additions of xanthan gum, respectively). (The different letters A–C marked in the bar chart indicated significant differences in the results of average particle size (*p* < 0.05); The different letters a–e marked in the line indicated significant difference in absolute value of Zeta potential (*p* < 0.05)).

The effects of the addition of AVE (I), chitosan (II), and xanthan gum (III) on the size of the Pickering emulsion oleogel and the absolute value of Zeta potential are shown in Figure 2. As shown in Figure 2I, with the increase in AVE level, the particle size of the Pickering emulsion oleogel showed a trend of first significantly decreasing and then increasing (*p* < 0.05). When the addition of AVE was 0%, the particle size of the oleogel was the largest, and when the addition of AVE was 0.1%, the particle size of the oleogel was the smallest. However, the absolute value of the Zeta potential of the oleogel showed a decreasing trend after a significant increase (*p* < 0.05). When the AVE addition was 0%, the absolute value of the Zeta potential of the oleogel was the smallest (<30), indicating that the addition of AVE could significantly improve the absolute value of the Zeta potential of the oleogel. When the AVE addition was 0%, the absolute value of zeta potential was at its maximum. It can be seen that the addition of AVE made the oleogel system more stable, which may have been due to the abundant flavonoids and polyphenols in AVE components and the fact that the structure of multiple hydroxyl groups can interact with the carbonyl groups of protein peptide bonds by forming hydrogen bonds [23]. However, when the amount of AVE increased further, it had side effects. When the AVE addition reached 0.1%, a stable oleogel system could be formed, and at this time, the system had the best dispersion state and the most stable structure.

From Figure 2II, with the increase in chitosan addition (1.0–2.5%), the particle size of the Pickering emulsion oleogel first decreased and then increased (*p* < 0.05), and the particle size of the Pickering emulsion oleogel was the smallest when the chitosan addition was 2.5%. This may have been because the increase in the added mass fraction of chitosan was conducive to the formation of a polysaccharide–protein complex between the polysaccharide complex and ovalbumin and the formation of an autoaggregate of chitosan, which increased its hydrophobicity, oil affinity, and particle stability [24]. When the addition of chitosan increased from 2% to 3%, the particle size of the oleogel increased. This may have been because the increase in mass fraction increased the particle size, because the high concentration of chitosan was not conducive to the stability of the particles, and the excess chitosan may have self-aggregated into larger particles [9]. The absolute Zeta potential trend of the oleogel was negatively correlated with the change in particle size, increasing first and decreasing gradually. When the addition of chitosan was 2.5%, the absolute Zeta potential of the oleogel had a larger value (greater than 45 mV), indicating that the addition of chitosan made the AVE–ovalbumin/chitosan/xanthan Pickering emulsion oleogel system better dispersed and more stable. Therefore, the addition of chitosan was selected to be 2.5%.

According to Figure 2III, with the increase in xanthan gum addition, the particle size of the composite system first gradually decreased and then significantly increased, while the absolute value of Zeta potential first increased and then decreased (*p* < 0.05). This showed that the addition of an appropriate amount of xanthan gum was conducive to the stability of the system, because xanthan gum had strong emulsifying stability and a high suspension stability effect, and the hardness and viscosity of the system were significantly increased [25]. However, the excessive addition of xanthan gum would have had a negative impact on the stability of the system, which may be because the viscosity of xanthan gum increased, resulting in insufficient emulsification of the cell crusher [26]. When the amount of xanthan gum was 0.3%, the oleogel particle size had the minimum value, and the absolute value of Zeta potential was the maximum. This indicated that the particle size distribution of the composite system was relatively uniform and stable when the addition of xanthan gum was 0.3%. This may be because the addition of xanthan gum increased the net charge of the emulsion drops, enhanced the electrostatic repulsion between the droplets, and increased the stability of the emulsion [27]. Single protein-based nanocapsules tend to aggregate under common food processing conditions, resulting in poor stability, while polysaccharides can inhibit the aggregation and precipitation of proteins by providing electrostatic repulsion and steric hindrance, making the system more environmentally sensitive [28]. Krstonošić et al. [27] also confirmed that the complex nanocarrier matrix formed by the interaction of protein and polysaccharide may help to further improve the protective effect and bioavailability of bioactive substances.

#### 2.2.2. Effects of Addition of AVE, Chitosan, and Xanthan Gum on Microstructure of Pickering Emulsion Oleogel

**Figure 3:**
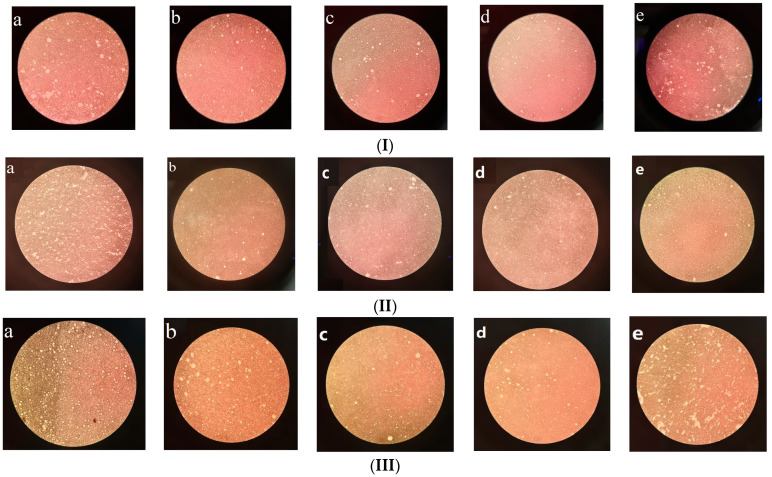
Effects of addition of AVE (**I**), chitosan (**II**), and xanthan gum (**III**) on microstructure of Pickering emulsion oleogel (200×). (In the figure (**I**), a–e represent 0, 0.05%, 0.1%, 0.15%, and 0.2% AVE additions, respectively. In the figure (**II**), a–e represent 1.0%, 1.5%, 2%, 2.5%, and 3% chitosan additions, respectively. In the figure (**III**), a–e represent 0%, 0.15%, 3%, 0.45%, and 0.6% xanthan gum additions, respectively.)

The effect of the addition of AVE (I), chitosan (II), and xanthan gum (III) on the microstructure of Pickering emulsion oleogel is shown in Figure 3. Through the observation of its microstructure (Figure 3I), we can see that AVE could significantly improve the droplet distribution of the oleogel. In the range of 0~0.1% AVE addition, the distribution of oil droplets gradually became uniform and stable with the increase in AVE addition amount. When AVE addition was 0.1%, the distribution of liquid droplets became more uniform and stable, with less aggregation and flocculation, but with the increase in AVE addition, the droplets also gradually became uneven, and many large aggregates were generated. This may have been due to the strong cross-linking between polyphenols/flavonoids and protein, which can affect the emulsification effect [23].

As shown in Figure 3II, with the increasing amount of chitosan (1~2.5%), the droplets of the oleogel system became fine and dispersed evenly. However, with the further addition of chitosan (2.5~3.0%), the homogeneity of the system decreased, and some droplets gathered, which may have been because chitosan was slightly soluble, and an excessive increase would lead to a poor dissolution effect. The effect of emulsification and dispersion was affected. Therefore, the addition of chitosan was determined to be 2.5%, which was basically consistent with the analysis results of particle size and Zeta potential.

The effect of different xanthan gum additions on the microstructure of the Pickering emulsion oleogel is shown in Figure 3III. When the addition of xanthan gum was 0%, there were a lot of flocculants in the composite system, as well as many aggregated droplets, and the dispersion was not uniform and not stable. With the addition of xanthan gum increasing from 0.15% to 0.3%, the droplets became smaller and more evenly dispersed. However, with the further addition of Xanthan gum (0.3~0.6%), the uniformity of the mixing system gradually deteriorated, and some large droplets were produced. It was speculated that the main reason was that the excessive addition of xanthan gum would make the system too thick, affecting the dissolution and emulsification of each component [25]. From the image, the homogeneity of sample c was relatively good, which also indicated that the stability of the system was relatively good when the amount of xanthan gum was 0.3%.

#### 2.2.3. Effects of Addition of AVE, Chitosan, and Xanthan Gum on Thermal Stability and Freeze–Thaw Stability of Pickering Emulsion Oleogel

**Figure 4:**
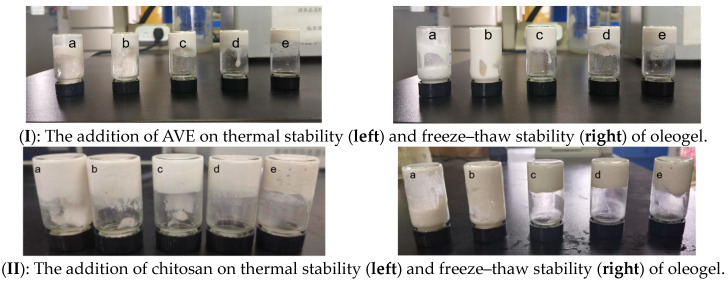

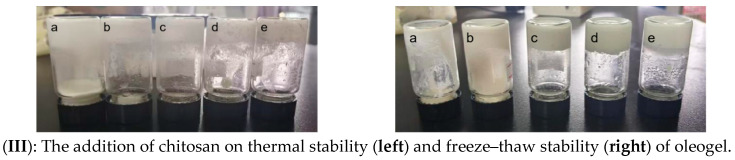
Effects of addition of AVE (**I**), chitosan (**II**), and xanthan gum (**III**) on thermal stability and freeze–thaw stability of Pickering emulsion oleogel. (In the figure (**I**), a–e represent AVE additions of 0, 0.05%, 0.1%, 0.15%, and 0.2%, respectively. In the figure (**II**), a–e represent chitosan additions of 1.0%, 1.5%, 2%, 2.5%, and 3%, respectively. In the figure (**III**), a–e represent xanthan gum additions of 0%, 0.15%, 3%, 0.45%, and 0.6%, respectively).

The effects of the addition of AVE (I), chitosan (II), and xanthan gum (III) on the thermal stability and freeze–thaw stability of the Pickering emulsion oleogel are shown in Figure 4. With the increase in AVE addition, the thermal stability and freeze–thaw stability of the oleogel were also improved. When the AVE content was less than 0.1%, the oleogel broke and deformed after heating, and obvious oil leakage could be observed. When the addition of AVE was greater than 0.1%, the heated oleogel was inverted and did not flow, showing certain plasticity (Figure 4I (left)). Similarly, after freeze-thawing, oil leakage occurred in samples with the addition of *Amomum villosum* Lour. less than 0.1%, and with the continuous increase in the amount of *Amomum villosum* Lour. added, the oleogel had good freeze-thawing stability (Figure 4I (right)). This indicated that the addition of AVE could significantly improve the thermal stability and freeze–thaw stability of the oleogel. It is speculated that the reason for this may be the complexation between AVE and protein, the change in physical and chemical properties, as well as the complex cross-linking between AVE, polysaccharide, and protein [23,29]. In summary, when the content of AVE was 0.1%, the AVE–ovalbumin/chitosan/xanthan gel Pickering emulsion oleogel had good thermal stability, freeze–thaw stability, and microstructure, which was consistent with the analysis results of particle size and Zeta potential.

As can be seen from Figure 4II (left), the thermal stability of the oleogel was improved with the increasing addition of chitosan in the range of 1~2.5%. When the added amount of chitosan was lower than 2%, the oleogel (a–c sample) broke and deformed, and obvious oil leakage occurred. However, when the addition of chitosan reached more than 2.5%, the oleogel after heating was inverted and did not flow and had certain plasticity. As can be seen from Figure 4II (right), after freeze-thawing, the oleogels of sample a and sample b with low added amounts of chitosan correspondingly had obvious oil leakage. When the mass fraction of chitosan reached more than 2%, the oleogels of sample c, d, and e all had relatively good freeze–thaw stability, among which, sample d had better stability from the image. This result indicated that the increase in chitosan can improve the thermal stability and freeze–thaw stability of the oleogel, which was speculated to be due to the fact that in addition to its network formation ability, chitosan can also adsorb on the ovalbumin interface to form a double-layer interface to enhance the interface stability [9]. Therefore, the strength of the interaction between chitosan and ovalbumin was crucial for the stability of the oleogel. In the emulsion, the interaction between chitosan and ovalbumin was mainly mediated by hydrogen bonds [30], which further improved the stability of the prepared oleogel.

As shown in Figure 4III (left), the thermal stability of the oleogel was correspondingly improved with the increasing amount of xanthan gum. When the amount of xanthan gum was lower than 0.3%, the oleogel cracked and deformed, and oil leakage occurred. When the addition of xanthan gum was 0.3~0.6%, after heating, the oleogel would not flow for a period of time, indicating that the addition of xanthan gum would enhance the thermal stability of the oleogel. As can be seen from Figure 4III (right), after freeze-thawing, oil leakage occurred in the oleogel group (samples a and b) with a small amount of xanthan gum added, and the oil leakage phenomenon was most serious in sample a without xanthan gum added. When the addition of xanthan gum reached more than 0.3%, the oleogel had good freeze–thaw stability. This indicated that the addition of xanthan gum could improve the thermal stability and freeze–thaw stability of the oleogel to a certain extent [25], and the appropriate addition of xanthan gum was 0.3~0.45%.

In conclusion, the particle size distribution of the AVE–ovalbumin/chitosan/xanthan Pickering emulsion oleogel were uniform and stable, and the dispersive state was the best when the addition of AVE was 0.1%, chitosan 2.5%, and xanthan gum 0.3~0.45%. Studies have shown that the properties of polyphenol–polysaccharide composite particles modified by non-covalent or covalent interaction between polyphenols and polysaccharides were better than those of polysaccharide–polysaccharide and polysaccharide–protein particles (covalent or non-covalent modification). This was because the polyphenol–polysaccharide complex adsorbed on the oil–water interface of a Pickering emulsion can form a physical barrier and inhibit the flocculation and coagulation of the emulsion. At the same time, polyphenols on the interface can remove free radicals generated during storage, thereby improving the physical and oxidative stability of the emulsion [31,32]. In addition, the polyphenol–polysaccharide complex can also confer other properties on Pickering emulsions, such as broad-spectrum antibacterial properties, controllable rheological properties, and digestive properties [33,34]. At present, some researchers have used the food-grade Pickering emulsion constructed with a polyphenol–polysaccharide complex to encapsulate and deliver the active substance, so as to improve the physical and chemical stability of the active substance and the digestible absorption rate in the human body.

### 2.3. Measurement of the Properties of AVE–Ovalbumin/Chitosan/Xanthan Pickering Emulsion Oleogel

#### 2.3.1. The Analysis of Rheological Properties

**Figure 5:**
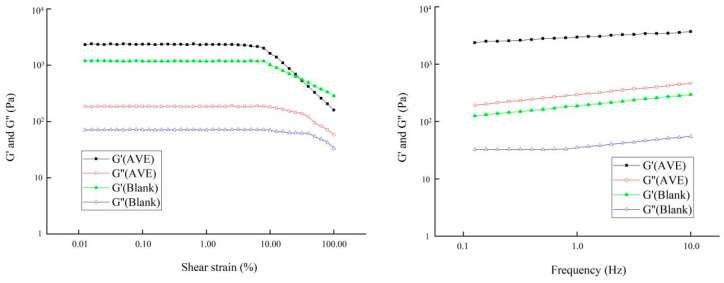
Strain sweep and frequency sweep curves of Pickering emulsion oleogels (AVE: the AVE-ovalbumin/chitosan/xanthan Pickering emulsion oleogel; Blank: the ovalbumin/chitosan/xanthan Pickering emulsion oleogel without AVE).

As can be seen from Figure 5, the G′ values of the AVE group and the blank group were always greater than G″, indicating that a gel structure had been formed, and the linear viscoelastic region of the oleogel was longer, indicating that its structure was not easily damaged by external forces and had good remodelability. When AVE was added to the oleogel system in the experimental group, G′ was significantly higher than that in the blank control group, indicating that the addition of AVE enhanced the gel properties of the oleogel. With the increase in frequency, the modulus value of the oleogel did not change significantly, indicating that the oleogel had good solid-like characteristics and the interior was composed of non-covalent physical cross-linking [35].

#### 2.3.2. The Analysis of Infrared Spectrum

**Figure 6:**
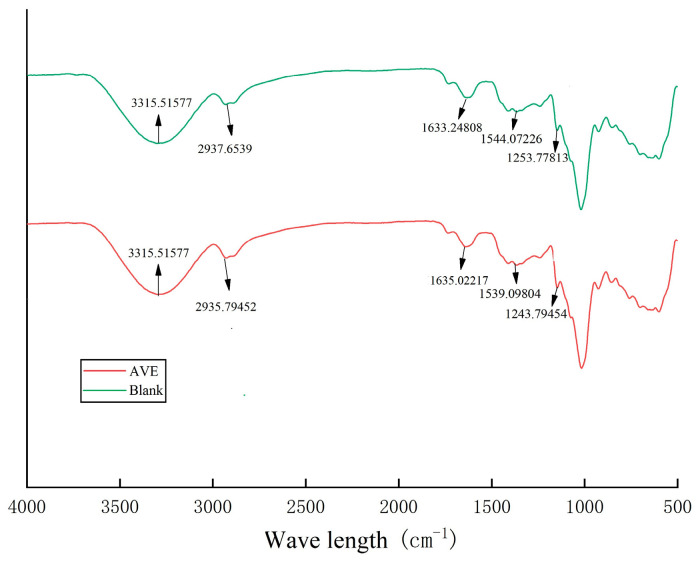
Fourier infrared spectrum of oleogel (AVE: the AVE–ovalbumin/chitosan/xanthan Pickering emulsion oleogel; blank: the ovalbumin/chitosan/xanthan Pickering emulsion oleogel without AVE).

Table 1 and Figure 6 show the Fourier infrared peak wavelength position and spectrum of the blank group and the experimental group (AVE) of oleogel, respectively. The acyl A band corresponded to the vibration of -NH and -OH, and the peak position and intensity were closely related to the hydrogen bond strength and the order of the spatial structure [36]. The weak absorption peak in the acyl B band was generated by the stretching vibration of the C-N bond. The amide I band was the characteristic spectrum band of the C=O and Schiff base C=N stretching wave, and the amide II band was mainly the reaction of the C-N stretching wave and N-H bending wave of proteins. The amide III band was mainly characterized by C-N stretching and N-H bending fluctuations of proteins [37,38]. The amide I band, amide II band, and amide III band could directly reflect the conformation of protein polypeptide chains, and their position changed directly, which reflected the changes in hydrogen bonds. When the characteristic band moved from a high wave number to a low wave number, it indicated that hydrogen bonds were forming. The more hydrogen bonds that were formed, the more obvious the change was [39].

As can be seen from Figure 6, the amide A band of the experimental group (AVE) widened and moved to a high wave peak, indicating that after the protein was combined with xanthan gum, chitosan, and AVE, the acyl A band binding between proteins was weakened. The intermolecular hydrogen bond binding together of the mixed system was weakened, which may have been due to the addition of AVE. The hydrogen bonding force between ovalbumin was weakened, which also corresponded to the increase in the absolute value of Zeta potential in the above results, and it was not easy to flocculate. Compared with the blank control group, the amide B band of the oil gel was slightly reduced, indicating that AVE was complexed with the protein, and the strength of the C-N bond between ovalbumin and polysaccharide (chitosan/xanthan gum) was reduced. By comparing the two groups of oleogels, the experimental group with AVE showed little change in the amide I band, while the amide II band and amide III band decreased to different degrees compared with the blank group, indicating that AVE may have complexed with ovalbumin and then coated with polysaccharide (chitosan/xanthan gum). The addition of AVE produced new hydrogen bonds with ovalbumin, chitosan, and xanthan gum, which strengthened the binding force between them and made the whole system more stable.

#### 2.3.3. The Analysis of Oxidative Stability

**Figure 7:**
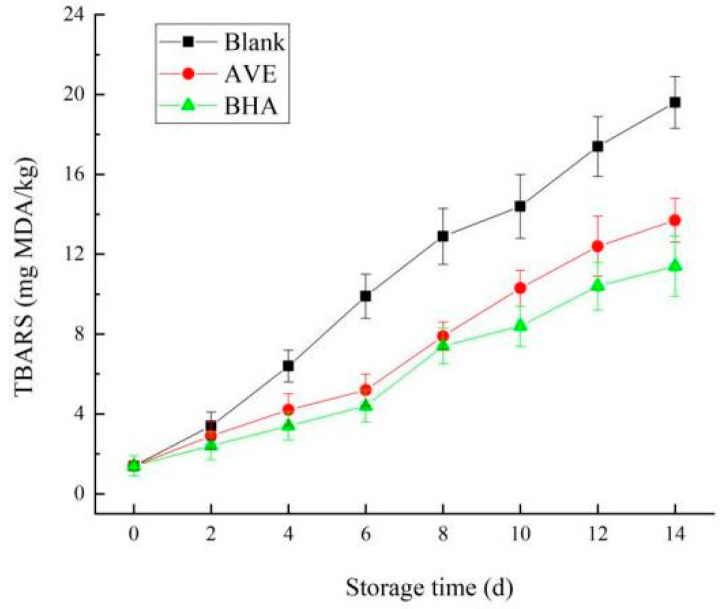
Changes in oleogel TBARS during storage (blank: the ovalbumin/chitosan/xanthan Pickering emulsion oleogel without AVE; AVE: the AVE–ovalbumin/chitosan/xanthan Pickering emulsion oleogel; BHT was the positive control).

In order to investigate the effect of AVE on the oxidation stability of oleogel, the oleogel without AVE was used as a blank control group, and the oleogel with 0.1% BHA as a positive control group (BHA group) was used to detect the oxidation of oleogel during 14 days of storage. As shown in Figure 7, with the extension of storage time, the TBARS of the three groups all showed an increasing trend, and the increasing rate of TBARS in the blank group was significantly higher than that in the AVE and BHA groups. It can be seen from the figure that the addition of AVE and BHA could more effectively delay the oxidation of oil in the oil gel system. On the whole, although the antioxidant effect of AVE was slightly inferior to that of BHA, the AVE group still showed an antioxidant effect close to that of BHA in the early stage of storage (0–8 d), but the MDA formation may have been accelerated in the later stage of storage (8~14 days) due to the continuous dissolution and oxidation of fats in the oleogel.

Vegetable oils, including soybean oil, generally contain a lot of unsaturated fatty acids, and although they are relatively healthy compared to saturated fatty acids, unsaturated fatty acids in fats and fats will automatically oxidize to produce primary oxidation products such as hydroperoxide. Although the polymer grid structure formed around the oil droplets of the oleogel can reduce the contact between the oil droplets and oxygen in the air to delay their oxidation process, the oxidation of unsaturated fatty acids still occurs [40]. AVE not only has many spillover effects on human health, but also is an effective natural antioxidant with a significant antioxidant effect, which can inhibit the oxidation process of fats and oils. In addition, the structure of polyphenols can also be complexed with proteins, strengthening the structural strength of polymer grids [41].

### 2.4. Application of AVE–Ovalbumin/Chitosan/Xanthan Gum Pickering Emulsion Oleogel in Cookies

#### 2.4.1. Effect of the Oleogels on Texture of the Cookies

**Figure 8:**
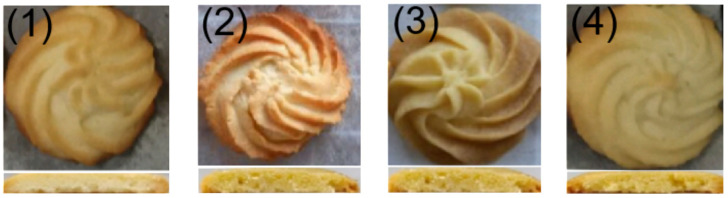
Appearance and cross-section of each sample ((**1**–**4**): cookies prepared with soybean oil, oleogel without AVE (blank), experimental oleogel (AVE), and butter, respectively).

**Figure 9:**
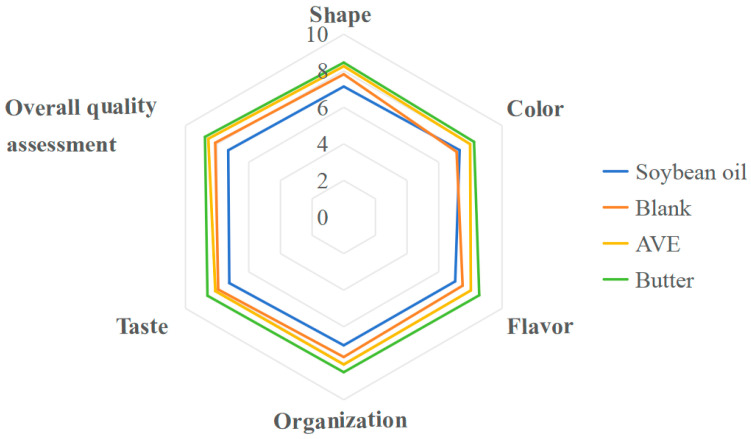
Sensory evaluation of cookies (soybean oil: cookies prepared with soybean oil; blank: cookies prepared with the oleogel, but no AVE; AVE: cookies prepared with the AVE–oleogel; butter: cookies prepared with butter).

The appearance and cross-section of each samples are shown in Figure 8, and the sensory score is shown in Figure 9. There were no significant differences in the scores of morphology, color, flavor, texture, taste, and overall quality assessment of the samples. Overall, the liquid soybean oil scored the lowest, the butter group scored the highest, and the blank group was lower than the AVE group, and although the AVE group did not score as high as the butter group, the difference was not significant.

**Figure 10:**
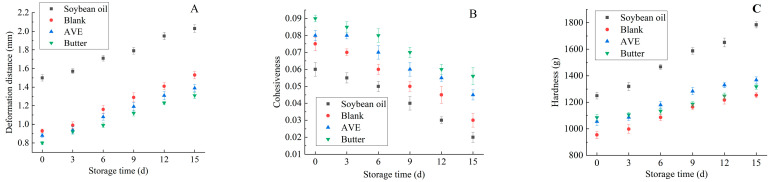
Effect of the oleogels on texture of cookies during storage. (**A**): Deformation distance, (**B**): cohesion, (**C**): hardness (soybean oil: cookies prepared with soybean oil; blank: cookies prepared with the oleogel, but no AVE; AVE: cookies prepared with the AVE–oleogel; butter: cookies prepared with butter).

**Figure 11:**
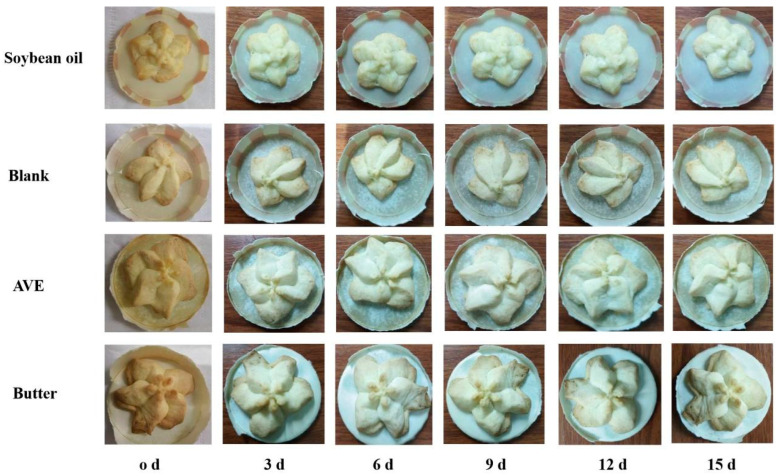
Effect of the oleogels on the appearance of the cookies during storage (soybean oil: cookies prepared with soybean oil; blank: cookies prepared with the oleogel, but no AVE; AVE: cookies prepared with the AVE–oleogel; butter: cookies prepared with butter).

In order to further evaluate the difference in the odd texture quality of different samples, the texture changes in cookies during storage were measured, and the results are shown in Figure 10 and Figure 11. The texture of a cookie is an important physical property. The greater the breaking force of the cookie, the higher the hardness; the larger the deformation distance before the cookie breaks, the lower the crispness; the stronger the relative resistance of the cookie to the second compression after the first compression deformation, the stronger the cohesiveness [42]. As can be seen from Figure 10 and Figure 11, with the extension of storage time, the deformation distance of samples in each group showed a gradually increasing trend, indicating that the brittleness of samples in each group gradually decreased, and the brittleness in the soybean oil group was lower than that in the blank group, AVE group, and butter group. At the same time, the cohesiveness of each group showed a downward trend (cohesiveness: butter group > AVE > blank > soybean oil), while the hardness of all kinds of samples increased gradually, and the hardness value of the soybean oil group was higher than that of AVE > butter > blank.

Compared with liquid oil (soybean oil), the AVE group had better brittleness and cohesiveness, but slightly weaker hardness. Compared with the blank control group, the AVE group had higher brittleness, cohesiveness, and hardness. Compared with solid fat (butter), the AVE group was slightly less brittle and cohesive than butter, but the hardness value was higher than butter. The reason for the above phenomenon may be that the liquid soybean oil was dispersed in the dough in the form of droplets, which could not cover all the flour surface, and the flour particles would contact with water to form gluten protein, which had poor ability to trap air [43]. However, oleogel gelated liquid oil into solid oil, which allowed sufficient air to enter and enhanced the ability of dough to trap air. Therefore, oil could be evenly wrapped on the surface of flour particles, which not only reduced the cross-linking of gluten, but also formed a limited gluten network structure and improved the ability to trap air [44,45]. At the same time, the addition of AVE could form a more stable spatial network structure and further improved the texture characteristics of the cookie, so as to form a crisp cookie.

In summary, although the texture properties of the AVE group (crispness, cohesion and hardness) were not as good as those of butter, the gap was not large, and they were far better than the blank group. This indicated that the ovalbumin/chitosan/xanthan oleogel prepared in this study had the potential to replace solid fat (butter) to a certain extent. In addition, the addition of AVE did not affect the texture and sensory properties of cookies, but also could delay the deterioration rate of the brittleness, cohesion, and hardness of cookies during storage. This was consistent with the research of Liu et al. [46], Jiang et al. [47], and Mao et al. [48].

#### 2.4.2. Effect of the Oleogels on Oil Oxidation of Cookies during Storage

**Figure 12:**
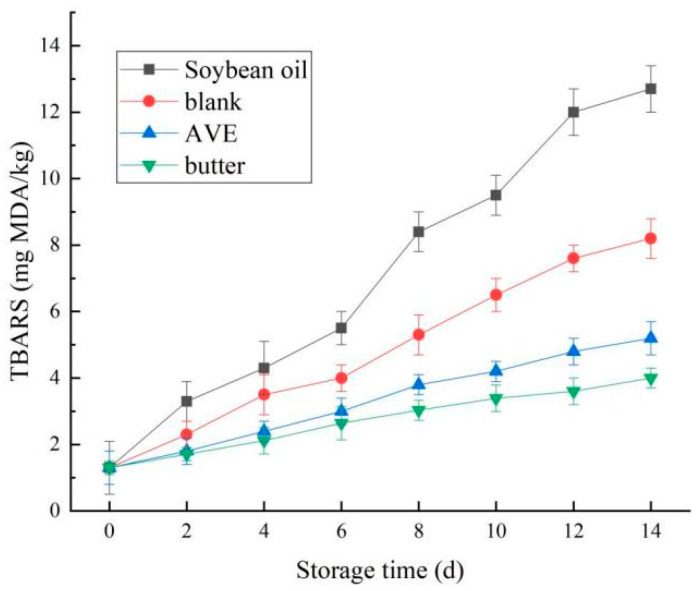
Effect of the oleogels on the TBARS of the cookies during storage (soybean oil: cookies prepared with soybean oil; blank: cookies prepared with the oleogel, but no AVE; AVE: cookies prepared with the AVE–oleogel; butter: cookies prepared with butter).

As shown in Figure 12, during the storage period of the 45 °C accelerated experiment, the TBARS of the four kinds of cookie products showed an increasing trend, and the oxidation stability showed significant differences. Among them, butter cookies had the highest oxidation stability, while soybean oil cookies had the lowest oxidation stability, which was consistent with the previous results. This was because liquid soybean oil had high unsaturated fatty acid content and was not protected by polysaccharide, so it was very easy to oxidize, while butter was rich in saturated fatty acids, so it had strong stability [49].

Compared with the blank group, the TBARS value of the AVE group increased slowly, which also indicated that the addition of AVE could delay the oxidation of lipids in cookies to a certain extent. This may have been due to the formation of a polymer network around the oil droplets, which enclosed the oil droplets and thus reduced the oil’s contact with oxygen, thus inhibiting the automatic oxidation of the oil [50]. The oxidation stability of the oleogel loaded with AVE was close to that of butter, which indicated that AVE prepared in this study can have good antioxidant activity in the AVE–ovalbumin/chitosan/xanthan Pickering emulsion oleogel, and it played an important role in the oil stability of cookies. Studies have shown that protein–polysaccharide–polyphenol terpolymer gels have good emulsifying ability and can significantly enhance the mechanical strength and antioxidant capacity of oleogels. Currently, they are used in saturated fat and hydrogenated fat substitutes, food texture improvers, and water-soluble/fat-soluble bioactive substance carriers. They have great potential in the fields of probiotic delivery, the co-delivery of health care products, solid lipid particles, essential oil systems, and microencapsulated oleogels [35].

## 3. Conclusions

AVE with high free radical scavenging capacity and extract yield was prepared, and when the addition of AVE, chitosan, and xanthan gum was 0.1%, 2.5%, and 0.3%, a uniform and stable AVE–ovalbumin/chitosan/xanthan Pickering emulsion oleogel was prepared. In addition, the addition of AVE could enhance the gel properties and solid-like properties of the oleogels, which was probably due to the new hydrogen bonds generated, which strengthened the binding force in the composite system, so that the whole system became more stable, even during storage. In addition, the application experiment results showed that the AVE–ovalbumin/chitosan/xanthan Pickering emulsion oleogel had similar sensory and texture properties (brittleness, cohesiveness, hardness) to the butter. The addition of AVE could delay the deterioration of the oil in the storage process of cookies. In summary, the AVE–ovalbumin/chitosan/xanthan Pickering emulsion oleogel was characterized with good structural and physicochemical stability, and AVE played an important role in the functional oleogel. This novel oleogel has great potential to be used in the future as a substitute for saturated fat (butter) in bakery products (cookies). At present, most fat substitutes can only partially replace saturated fat, and their effects on food digestion characteristics and mechanisms of action need to be further clarified. The bioavailability of complex systems and their metabolic and absorption processes in vivo need to be further explored.

## 4. Materials and Methods

### 4.1. Material

*Amomum villosum* Lour. was provided by Duomai (Fujian) Food Co., Ltd. (Quanzhou, China). Ovalbumin (from chicken egg whites, ≥80% (agarose gel electrophoresis)), chitosan (N-degree of deacetylation ≥ 90%, the relative molecular mass is about 50–90 kDa), and xanthan gum (the average molecular weight is about 2 × 10^6^~2 × 10^7^), all food grade, were purchased from Zhejiang Yinuo Biotechnology Co., Ltd. (Lanxi City, China) Ethanol, thiobarbituric acid, trichloroacetic acid, hydrochloric acid, and malondialdehyde, all analytically pure, were purchased from Xilong Science Co., Ltd. (Shantou City, China). ORAC kits were purchased from Nanjing Jiancheng Bioengineering Research Institute Co., Ltd. (Nanjing City, China) Soybean oil, butter, low-gluten flour, milk, sugar, salt, and eggs were purchased from the Zhangzhou RT-Fat supermarket.

### 4.2. Preparation and Optimization of AVE

#### 4.2.1. Preparation of AVE

*Amomum villosum* Lour. was cleaned and then dried in a drying oven at 50 °C for 24 h. The dried *Amomum villosum* Lour. was crushed into powder and was placed in the refrigerator for later use. According to the liquid–solid ratio of 40 mL/g, the ethanol solution with a 60% volume fraction was added to the cell crusher for ultrasound pretreatment for 1.5 min, the ultrasonic power was 240 W, and the ultrasound was stopped for 2 s every 4 s. The treated mixed solution was extracted in a water bath at 60 °C for 2 h. The mixed system was centrifuged at 4000 r/min for 15 min, and then, the supernatant was collected. The paste extract was obtained after vacuum evaporation at 45 °C, and the AVE was obtained after vacuum freeze-drying (−50 °C, 48 h) and stored at −20 °C for later use.

#### 4.2.2. The Optimization of the AVE Extraction Process

The effects of different ultrasonic processing times of 0.5, 1, 1.5, 2, and 2.5 min; different ethanol solution concentrations of 40%, 50%, 60%,70%, and 80%; different liquid–solid ratios (the volume of ethanol solution: the quality of AVE) of 20, 30, 40, 50, and 60 mL/g; different water bath temperatures of 40, 50, 60, 70, and 80 °C; and different water bath times of 1, 1.5, 2, 2.5, and 3 h on the free radical scavenging rate and yield of AVE were investigated.

#### 4.2.3. Free Radical Scavenging Capacity of AVE

Following the ORAC kit method, AVE solutions of 100, 120, 140, 160, 180, and 200 μg/mL, were prepared with 75 mmol/L phosphoric acid buffer (pH = 7.4) with 10 mL, respectively. The sodium fluorescein (2.1 μmol/L) and 2,2′-diisobutylamidine dihydrochloride (AAPH) (100 mmol/L) were prepared with 75 mmol/L phosphoric acid buffer (pH = 7.4) too. We added 1 mL of sodium fluorescin and a 1 mL sample solution in a test tube and held them in a 37 °C water bath for 20 min. Then, we added 1 mL of AApH at 100 mmol/L, and the fluorescence intensity was measured every 1 min (the excitation wavelength was 485 nm, the emission wavelength was 535 nm for determination), and the continuous determination took place for 90 min. After the determination, the fluorescence intensity–time curve was calculated, that is, the area under the fluorescence decay curve (AUCSample). The other two groups were the fluorescence decay curve area (AUCFL) of the group with only sodium fluorescein and phosphate buffer added and the fluorescence decay curve area (AUCAAPH) of the group with buffer and sodium fluorescein and AAPH added. The formula for calculating the inhibition rate was as follows:

(1) $$Free\;radical\;scavenging\;capacity\;(\%)=\frac{{AUC}_{sample}-{AUC}_{AAPH}}{{AUC}_{FL}-{AUC}_{AAPH}}\times 100$$

Among them, AUC_Sample_ was the area under the fluorescence attenuation curve. AUC_FL_ was the fluorescence decay curve area of the experimental group with only sodium fluorescein and phosphate buffer added, and AUC_AAPH_ was the fluorescence decay curve area of the group in which buffer, sodium fluorescein, and AAPH were added.

#### 4.2.4. The Yield of AVE

The ratio of the weight of the mixture after extraction and vacuum freeze-drying to the weight of the *Amomum villosum* Lour. powder was denoted as the yield of AVE.

### 4.3. The Preparation of Ovalbumin/Xanthan Gum/Chitosan Pickering Emulsion Oleogel Added with AVE

We added 0.15 g of ovalbumin to 15 mL of deionized water, stirred it thoroughly until it was completely dissolved, and sealed it overnight and fully hydrated the ovalbumin to obtain a 1% ovalbumin solution. Then, AVE was added and fully dissolved so that its final concentration was 0 to 0.2%. After that, xanthan gum and chitosan were added (the final concentrations of the two were 0~0.6% and 1~3%, respectively) and fully stirred until they were completely dissolved, and the protein–polysaccharide composite particles were configured. Finally, 35 mL of soybean oil was added to the composite system. The soybean oil was added twice and emulsified at high speed in an ultrasonic cell crusher (VCX800) (SONICS&MATERIALS, Newtown, Connecticut, USA) for 2 min each time. Finally, the Pickering emulsion oleogel was obtained [51].

The effects of AVE addition (0, 0.05%, 0.1%, 0.15%, 0.2%), chitosan addition (1%, 1.5%, 2%, 2.5%, 3%), and xanthan gum addition (0, 0.15%, 0.3%, 0.45%, 0.6%) on the particle size, Zeta potential, thermal stability, freeze–thaw stability, and microscopic morphology of the oleogel were investigated.

### 4.4. Determination of Physicochemical Properties of Pickering Emulsion Oleogels

#### 4.4.1. Determination of Particle Size and Zeta Potential

The average particle size of the Pickering emulsion oleogel was determined by laser diffraction using a Malvern laser particle size analyzer (Zetaszier Nano-ZS90) (Malvern Instruments Co., Ltd., Malvern, UK). The sample was added to the circulation tank until the shading reached about 12% for determination.

The Zeta potential was measured with the Malvern nanoparticle analyzer, the sample was diluted 100 times, the sample dilution was absorbed into the Zeta potential pool, and the Zeta potential pool was put into the nanoparticle particle size analyzer to determine the Zeta potential. Each sample was measured 3 times, and the results were averaged.

#### 4.4.2. Observation of Microstructure

The microstructure of the oleogel was observed using a biological digital microscope (DM500-ICC50W) (Leica Corporation, Wetzlar, Germany). An appropriate amount of the oleogel was placed on a dry and clean slide, and the slide was covered to make the sample evenly distributed. Then, the samples were observed under the ordinary light of a 10× eyepiece and 20× objective lens, and the microstructure of the oleogel was photographed.

#### 4.4.3. Determination of Thermal Stability and Freeze–Thaw Stability

The sealed sample of the Pickering emulsion oleogel was placed in a boiling water bath for 15 min, and then cooled to room temperature immediately in an ice bath, and then, the appearance and microstructure of the oleogels after treatment were observed, and their thermal stability and freeze–thaw stability were measured [51].

#### 4.4.4. Measurement of Rheological Properties

An appropriate amount of Pickering emulsion oleogel was placed on the sample table with a plate with a diameter of 40.0 mm, the plate spacing was set at 1 mm, the shear frequency was constant at 1 Hz, the test temperature was 25 °C, and the linear viscoelastic region of the oleogel was determined by strain scanning at the shear strain of 0.01~100.00%. The shear strain value in the linear viscoelastic region was constant, and the frequency scanning was performed at the frequency of 0.1~10.0 Hz to obtain the change curves of the elastic modulus (G′) and viscous modulus (G″) of the gel oil.

#### 4.4.5. Analysis of Infrared Spectrum

A Fourier Transform Infrared Spectrometer (iS™ 20, ThermoFisher, Waltham, MA, USA) was used for total reflection scanning analysis. The oleogel system was freeze-dried under vacuum, and the samples of 3 mg to be tested were mixed with 200 mg of potassium bromide and ground. The samples were scanned 32 times in the frequency range of 4000–500 cm^−1^ with a resolution of 4 cm^−1^.

#### 4.4.6. Determination of Oxidation Stability

First, 10 g of freshly prepared oleogels was placed in a 20 mL serum vial and cultured in an incubator at 40 °C for 14 days to accelerate lipid oxidation. The content of secondary oxidation products (malondialdehyde) was measured every two days, and the results were expressed as thiobarbiturate values (TBARS).

The TBARS value was determined by Wan et al.’s [51] method: The thiobarbituric acid reagent was prepared by dissolving 0.375 g of thiobarbituric acid (0.375%, *w*/*v*) and 15 g of trichloroacetic acid (15%, *w*/*v*) in 100 mL of 0.25 M hydrochloric acid. Then, 4 mL of thiobarbituric acid reagent was added to 0.5 g of oleogel. The mixture was heated in a boiling water bath for 15 min and immediately cooled to room temperature under running water. The resulting mixture was centrifuged at 8000× *g* rpm for 15 min. After centrifugation, the supernatant was taken and the absorbance was measured at 532 nm, and the result was expressed as mg MDA/kg sample.

### 4.5. Application of Oleogels in Cookies

#### 4.5.1. The Making of Cookies

The cookie recipe was 50 g of fat, 15 g of caster sugar, 15 g of powdered sugar, 40 g of egg, 80 g of gluten powder, and 1 g of salt. Among them, soybean oil, oleogel with/without AVE, and butter were used to make cookies. The production method was as follows: Firstly, the grease was put into the mixing tank and beaten for 3 min. The caster sugar and icing sugar were added in the mixture and continued to cook for 5 min. Secondly, the beaten egg mixture was added in batches and beaten well. The plain flour and salt were sifted into the mixture well to form a dough. Then, the dough was divided into small pieces of dough with a mass of 15 g and squeezed into a specific cookie shape with a piping nozzle. Finally, the cookies were put in the oven, with the maximum heat being 190 °C and the minimum heat being 190 °C, and baked for 20 min. After cooling, the cookies were packed into a lid and stored at room temperature until analysis.

#### 4.5.2. The Texture Analysis of Cookies

The texture profiling of the cookies was performed with a texture analyzer (CT3-10K) (Bollefield, MA, USA). Using the TA-7 probe, two-step compression of the sample was performed at 0.5 mm/s, and two-step compression was performed at 3 mm from the center of the cookie. The measured forces in these two cycles were plotted as a function of time to obtain the TPA curve of the sample. The samples were tested every 3 days.

#### 4.5.3. The Oil Oxidation of Cookies

The cookies were stored in an oven at 45 °C for the accelerated experiment. The degree of grease oxidation in cookies was expressed by the TBARS value. The determination method was the same as “4.4.6”.

#### 4.5.4. The Sensory Evaluation of Cookies

According to the scoring criteria in Table 2, the sensory evaluation of cookies prepared with different kinds of fats was carried out. The different products were numbered randomly and distributed to ten sensory evaluators, who were trained and experienced in sensory evaluation panels, with a 1:1 ratio of male to female. The attributes of shape, color, flavor, organization, taste, and overall quality assessment of the cookie samples were evaluated. The total score of each attribute was 9 points.

### 4.6. Statistical Analysis of Data

All experimental tests were repeated 3 times, and the data were expressed as mean ± standard deviation. Origin 2022 was used for mapping, SPSS 22.0 was used for data processing, Duncan’s multiple comparison was performed for analysis of the significance of the data, and the data were expressed as mean ± standard deviation. *p* < 0.05 indicated a significant difference between data.

## Figures and Tables

**Table 1:** Position of peak wavelength in infrared spectra of oleogels.

Samples	Wavelength (cm^−1^)				
	Amide A Band	Amide B Band	Amide I Band	Amide II Band	Amide III Band
Blank	3315.51577	2937.6539	1633.24808	1544.07226	1253.77813
AVE	3315.51577	2935.79452	1635.02217	1539.09804	1243.79454

Note: Blank: the ovalbumin/chitosan/xanthan Pickering emulsion oleogel without AVE; AVE: the AVE–ovalbumin/chitosan/xanthan Pickering emulsion oleogel.

**Table 2:** Sensory evaluation criteria for cookies.

Attributes	Scoring Criteria	Total Score
Shape	The shape was a complete shape, uniform in size and thickness (7–9 points)	9 points
	The shape was relatively complete, and the size and thickness were basically uniform (4–6 points)	
	Incomplete shape and deformation, and uneven thickness (1–3 points)	
Color	The overall color of the surface was yellow, without overfocal or white phenomena (7–9 points)	9 points
	The surface color was more uniform, dark yellow, or light white (4–6 points)	
	Uneven surface color, and a large number of overfocal or white phenomena (1–3 points)	
Flavor	Baking aroma, no odor (7–9 points)	9 points
	Strong baking smell, basically no odor or slight odor (4–6 points)	
	Weak baking flavor, strong odor (1–3 points)	
Texture	The internal texture showed a uniform and dense porous texture, basically without holes (7–9 points)	9 points
	The interior presented a relatively uniform porous texture, with small holes (4–6 points)	
	The internal porous texture was uneven and dense, with large holes (1–3 points)	
Taste	Crispy, delicate, and not sticky (7–9 points)	9 points
	Crispy, delicate, and slightly sticky (4–6 points)	
	Not crisp, not delicate, and stuck to teeth (1–3 points)	
Overall quality assessment	Overall quality assessment was very good (7–9 points)	9 points
	Overall quality assessment was generally liked (4–6 points)	
	Overall quality assessment was bad (1–3 points)	

## Data Availability

The original contributions presented in the study are included in the article; further inquiries can be directed to the corresponding author.

## References

[1] Pehlivanoğlu H., Demirci M., Toker O.S., Konar N., Karasu S., Sagdic O. (2018). Oleogels, a promising structured oil fordecreasing saturated fatty acid concentrations: Production and food-based applications. Crit. Rev. Food Sci. Nutr..

[2] Zhu Y., Bo Y., Liu Y. (2019). Dietary total fat, fatty acids intake, and risk of cardiovascular disease: Adose-response meta-analysis of cohort studies. Lipids Health Dis..

[3] Chowdhury B., Sharma A., Akshit F.N.U., Mohan M.S., Salunke P., Anand S. (2023). A review of oleogels applications in dairy foods. Crit. Rev. Food Sci..

[4] Feichtinger A., Scholten E. (2020). Preparation of protein oleogels: Effect on structure and functionality. Foods.

[5] Patel A., Dewettinck K. (2016). Edible oil structuring: An overview and recent updates. Food Funct..

[6] Li J., Jin W., Xu W., Liu G., Huang Q., Zhu Z., Li S.Y., Cheng S.Y. (2020). Effect of charge density of polysaccharide on self-assembly behaviors of ovalbumin and sodium alginate. Int. J. Biol. Macromol..

[7] Vicente J., Pereira L.J.B., Bastos L.P.H., Carvalho L.P., GeraldoGarcia-Rojas M., Elard E. (2018). Effect of xanthan gum or pectin addition on Sacha Inchi oil-in-water emulsions stabilized by ovalbumin or tween 80: Droplet size distribution, rheological behavior and stability. Int. J. Biol. Macromol..

[8] Niu F., Dong Y., Shen F., Wang J., Liu Y.T., Su Y., Xu R., Wang J., Yang Y. (2015). Phase separation behavior and structural analysis of ovalbumin-gum arabic complex coacervation. Food Hydrocoll..

[9] Tang Y., Gao C., Zhang Y., Tang X.Z. (2022). A review of literature on Pickering emulsion gels stabilized by polysaccharide-based particles. Food Sci..

[10] Huang Z.Z., Yang X.X., Chen Q.Q., Chen L.Q., Liang S.Y., Zeng Q.Z., Zhang R.F., Huang F., Dong L.H., Su D.X. (2022). Ferulic acid and EGCG alter the structural characteristics of ovalbumin and its application in mineral loading. Int. J. Food Sci. Technol..

[11] Huang Z.Z., Yang X.X., Liang S.Y., Chen L.Q., Dong L.H., Rahaman A., He S., Shen Y.B., Su D.X. (2022). Polysaccharides improved the viscoelasticity, microstructure, and physical stability of ovalbumin-ferulic acid complex stabilized emulsion. Int. J. Biol. Macromol..

[12] Abu Zarim N., Zainul Abidin S., Ariffin F. (2021). Shelf life stability and quality study of texture-modified chicken rendang usingxanthan gum as thickener for the consumption of the elderly with dysphagia. Food Biosci..

[13] Zhu J.Z., Liu L., Li X.T., Zhang Q., Wang Z., Chen N., Wang H., Xie F., Qi B., Jiang L.Z. (2024). Construction of soybean oil bodies-xanthan gum composite oleogels by emulsion-templated method: Preparation, characterization, and stability analysis. Food Hydrocoll..

[14] Sagis L., Scholten E. (2014). Complex interfaces in food: Structure and mechanical properties. Trends Food Sci. Technol..

[15] Veardo V., Ferioli F., Riciputi Y., Iafelice G., Marconi E., Caboni M.F. (2009). Evaluation of lipid oxidation in spaghetti pasta enriched with long chain n-3 polyunsaturated fatty acids under different storage conditions. Food Chem..

[16] Feng L.L., Wang Z.C., Lei Z.W., Zhang X.F., Zhai B.T., Sun J., Guo D.Y., Wang D., Luan F., Zou J.B. (2024). *Amomum villosum* Lour.: An insight into ethnopharmacological, phytochemical, and pharmacological overview. J. Ethnopharmacol..

[17] Cao G.H., Zhang X., Wang X.F., Xiong J., Wang J., Li X.G., Xiao J.B., Zhao R.H., He S. (2020). Comparison of bacteriostatic effects of different extracts from Fructus amomi and its salt-processed products. Sci. Technol. Food Ind..

[18] Yip K.M., Xu J., Tong W.S., Zhou S.S., Yi T., Zhao Z.Z., Chen H.B. (2016). Ultrasound-assisted extraction may not be a better alternative approach than conventional boiling for extracting polysaccharides from herbal medicines. Molecules.

[19] Jovanović A.A., Đorđević V.B., Zdunić G.M., Pljevljakušić D.S., Šavikin K.P., Gođevac D.M., Bugarski B.M. (2017). Optimization of the extraction process of polyphenols from *Thymus serpyllum* L. herb using maceration, heat- and ultrasound-assisted techniques. Sep. Purif. Technol..

[20] Yu X.F., Wang J.H., Zhang M., Ma X.F., Xu Q.Q. (2024). Green and efficient extraction of polyphenols from the leaves of Quercus dentata Thunb and in vitro antioxidant activities of polyphenol extract. Sustain. Chem. Pharm..

[21] Rodríguez-Aguilar F., Eugenia Ortega-Regules A., Ramírez-Rodrigues M.M. (2024). Influence of time-temperature in the antioxidant activity, anthocyanin and polyphenols profile, and color of Ardisia compressa K. extracts, with the addition of sucrose or citric acid. Food Chem..

[22] Yan Y.J., Li X., Wan M.J., Chen J.P., Li S.J., Cao M., Zhang D.Y. (2015). Effect of extraction methods on property and bioactivity of water-soluble polysaccharides from *Amomum villosum*. Carbohydr. Polym..

[23] Li H.L., Zhou X.L., Wu X.J., Wu W. (2024). Advances in the study of polyphenol regulation of lipid-protein co-oxidation in emulsions of proteins, polysaccharides and protein-polysaccharide complexes. Food Sci..

[24] Kavya M., Udayarajan C., Fabra M.J., López-Rubio A., Nisha P. (2024). Edible oleogels based on high molecular weight oleogelators and its prospects in food applications. Crit. Rev. Food Sci. Nutr..

[25] Abdollahi M., Goli S.A.H., Soltanizadeh N. (2020). Physicochemical properties of foam-templated oleogel based on gelatin and xanthan gum. Eur. J. Lipid Sci. Technol..

[26] Yan Y.Z., Chen S.L., Deng L., Duan Y.X., Huang Z.H., Gong D.M., Zhang G.W. (2024). Construction and characterization of egg white protein -gallic acid-xanthan gum-based emulsion and oleogel. Food Hydrocoll..

[27] Krstonošić V., Pavlović N., Nikolić I., Milutinov J., Ćirin D. (2024). Physicochemical properties and stability of oil-in-water emulsions stabilized by soy protein isolate and xanthan gum. Int. J. Biol. Macromol..

[28] Gan C.F., Liu Q., Zhang Y., Shi T.Y., He W.S., Jia C.S. (2022). A novel phytosterols delivery system based on sodium caseinate-pectin soluble complexes: Improving stability and bioaccessibility. Food Hydrocoll..

[29] Zhang H.D., Yang X.Y., Zhong R.B., Huo Y.M., Zhu Y.J., Liang P. (2022). Antioxidative properties of fish roe peptides combined with polyphenol on the fish oil oleogel. J. Sci. Food Agric..

[30] Yang W., Xu C., Liu F., Sun C.X., Yuan F., Gao Y.X. (2015). Fabrication mechanism and structural characteristics of the temnaryaggregates by 1 actofenin, pectin, and- epig allo catechin gallate using multisp ectroscopicmethods. J. Agric. Food Chem..

[31] Zhang G.G., Zheng C.M., Huang B.Q., Fei P. (2020). Preparation of acylated pectin with gallic acid through enzymatic method and their emulsifying properties, antioxidation activities and antibacterial activities. Int. J. Biol. Macromol..

[32] Shahbazi M., Jäger H., Ettelaie R. (2021). Development of an antioxidative pickering emulsion gel through polyphenol-inspired free-radical grafting of microcrystalline cellulose for 3D food printing. Biomacromolecules.

[33] Li Q., Chen P., Li Y., Li B., Liu S.L. (2020). Construction of cellulose-based Pickering stabilizer as a novel interfacial antioxidant: A bioinspired oxygen protection strategy. Carbohydr. Polym..

[34] Wang R., Zhou J. (2022). Waxy maize starch nanoparticles incorporated tea polyphenols to stabilize Pickering emulsion and inhibit oil oxidation. Carbohydr. Polym..

[35] Cui X.T., Saleh A.S.M., Yang S., Wang N., Wang P., Zhu M.P., Xiao Z.G. (2023). Oleogels as animal fat and shortening replacers: Research advances and application challenges. Food Rev. Int..

[36] Shang J.G., Zhong F., Zhu S., Huang D.J., Li Y. (2021). Formation, structural characteristics and physicochemical properties of beeswax oleogels prepared with tea polyphenol loaded gelators. Food Funct..

[37] Hashim D.M., Man Y.B.C., Norakasha R., Shuhaimi M., Salmah Y., Syahariza Z.A. (2010). Potential use of Fourier transform infrared spectroscopy for differentiation of bovine and porcine gelatins. Food Chem..

[38] Muyonga J.H., Cole C.G.B., Duodu K.G. (2004). Fourier transform infrared (FTIR) spectroscopic study of acid soluble collagen and gelatin from skins and bones of young and adult Nile perch (*Lates niloticus*). Food Chem..

[39] Su Y.R., Tsai Y.C., Hsu C.H., Chao A.C., Lin C.W., Tsai M.L., Mi F.L. (2015). Effect of grape seed proanthocyanidin-gelatin colloidal complexes on stability and in vitro digestion of fish oil emulsions. J. Agric. Food Chem..

[40] Zhao W.J., Wei Z.H., Xue C.H., Meng Y. (2023). Development of food-grade oleogel via the aerogel-templated method: Oxidation stability, astaxanthin delivery and emulsifying application. Food Hydrocoll..

[41] Chen Y., Li Z.S., Yi X.Z., Kuang H.R., Ding B.M., Sun W.Q., Luo Y.C. (2020). Influence of carboxymethylcellulose on the interaction between ovalbumin and tannic acid via noncovalent bonds and its effects on emulsifying properties. LWT.

[42] Li S.Y., Wu G.C., Li X.J., Jin Q.Z., Wang X.G., Zhang H. (2021). Roles of gelator type and gelation technology on texture and sensory properties of cookies prepared with oleogels. Food Chem..

[43] Jang A., Bae W., Hwang H., Lee H.G., Lee S.Y. (2015). Evaluation of canola oil oleogels with candelilla wax as an alternative to shortening in baked goods. Food Chem..

[44] Jacob J., Leelavathi K. (2007). Effect of fat-type on cookie dough and cookie quality. J. Food Eng..

[45] Mert B., Demirkesen I. (2016). Reducing saturated fat with oleogel/shortening blends in a baked product. Food Chem..

[46] Liu T., Zheng J., Du J., He G.S. (2024). Food processing and nutrition strategies for improving the health of elderly people with dysphagia: A review of recent developments. Foods.

[47] Jiang Q.Y., Zhang M., Mujumdar A.S. (2021). Novel evaluation technology for the demand characteristics of 3D food printing materials: A review. Crit. Rev. Food Sci..

[48] Miao W.B., Fu Y.J., Zhang Z.H., Lin Q.Z., Li X.J., Sang S.Y., McClements D.J., Jiang H., Ji H.Y., Qiu C. (2024). Fabrication of starch-based oleogels using capillary bridges: Potential for application as edible inks in 3D food printing. Food Hydrocoll..

[49] Sinha S.S., Upadhyay A., Singh A. (2024). Development and optimization of oleogel made with soy protein isolate and xanthan gum using emulsion template approach and its comparison with solid fats. Heliyon.

[50] Liu F.G., Mcclements D.J., Ma C.C., Liu X.B. (2023). Novel colloidal food ingredients: Protein complexes and conjugates. Annu. Rev. Food Sci. Technol..

[51] Wan Z., Zhao H.Y., Zeng C.X., Xia H.P., Guo S.Y., Tang C.L. (2021). Characterization and application of rapeseed oil-low erucic acid oleogel based on Pickering emulsion. J. Hunan Agric. Univ. (Nat. Sci.).

